# Routine urinalysis for early clinical assessment in patients with vegetation-like echocardiographic findings

**DOI:** 10.1186/s12879-026-14072-1

**Published:** 2026-07-23

**Authors:** Kilyoon Pack, Kyung Eun Ha, Taeil Yang, Wook-Jin Chung, Mi-Seung Shin, Jeonggeun Moon

**Affiliations:** 1 Department of Critical Care Medicine, Samsung Medical Center, Sungkyunkwan University School of Medicine, Seoul, Republic of Korea; 2 Division of Cardiology, Department of Internal Medicine, Gil Medical Center, Gachon University College of Medicine, 21 Namdong-daero 774beon-gil, Namdong- gu, Incheon, 21565 Republic of Korea

**Keywords:** Endocarditis, Urinalysis, Echocardiography

## Abstract

**Background:**

Incidental vegetation-like findings on echocardiography prompt extensive diagnostic evaluation for infective endocarditis (IE), even in patients with low clinical suspicion. Given the association between IE and renal involvement, we evaluated whether routine urinalysis could serve as a simple adjunctive tool to aid early clinical assessment in this setting.

**Methods:**

We retrospectively analyzed adults with vegetation-like echocardiographic findings at a tertiary center between 2010 and 2024. Patients were classified as those with confirmed IE [IE (+)] and those in whom IE was ultimately excluded [IE (–)]. Urinalysis parameters at presentation were analyzed, and a simple additive score was constructed.

**Results:**

A total of 459 patients were included, comprising 202 with IE (+) and 257 with IE (–). Proteinuria, hematuria, and pyuria were more frequent in IE (+) and remained independently associated with IE in multivariable analysis. An additive score incorporating these variables (1 point each; range 0–3) demonstrated acceptable discriminatory ability (area under the curve, 0.77; 95% CI, 0.72–0.81). A score of 0 showed a relatively high sensitivity (78%) with acceptable specificity (67%).

**Conclusions:**

The absence of hematuria, proteinuria, and pyuria on routine urinalysis may serve as a supportive adjunct—not a rule-out test—for early clinical assessment.

**Supplementary Information:**

The online version contains supplementary material available at https://doi.org/10.1186/s12879-026-14072-1.

## Background

Incidental intracardiac masses are identified during routine examinations in patients with limited clinical context for etiologic assessment [1, 2]. Establishing whether such masses are of infectious or noninfectious etiology represents a clinically important diagnostic challenge [3, 4]. Vegetation-like findings on echocardiography frequently drive extensive diagnostic evaluation, including multimodality imaging or tissue biopsy [5, 6], even in patients without overt signs of infection, reflecting the lack of reliable strategies to confidently exclude an infectious etiology at an early stage [7–10].

Current diagnostic frameworks for infective endocarditis (IE) emphasize microbiological confirmation and imaging when clinical suspicion is high but provide limited guidance for patients with equivocal echocardiographic findings and low pretest probability [11, 12]. IE is often associated with immune complex–mediated renal involvement, manifesting as hematuria, proteinuria, or pyuria on routine urinalysis [13–15]. However, its application as a practical decision-support tool in early clinical assessment, particularly in patients with equivocal echocardiographic findings, has not been systematically explored. Given its low cost and widespread availability, we evaluated whether routine urinalysis may serve as a simple adjunctive tool to support early clinical triage in patients with vegetation-like echocardiographic findings.

## Methods

### Study population

**Fig. 1:**
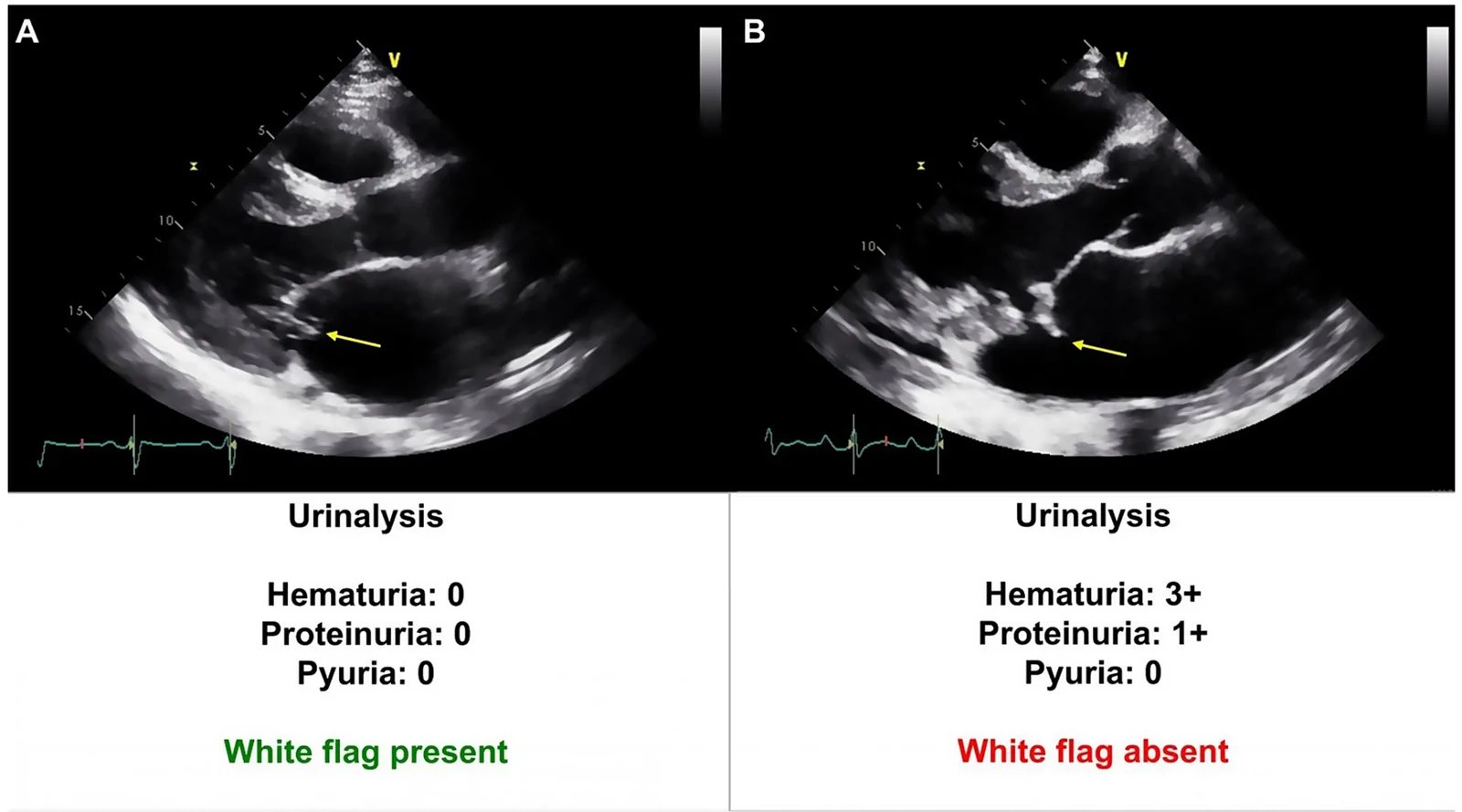
Representative echocardiographic and urinalysis findings illustrating the “White Flag Sign.”. (**A**) A patient with vegetation-like findings on transthoracic echocardiography (yellow arrow) who was ultimately classified as not having infective endocarditis. Routine urinalysis showed no hematuria, proteinuria, or pyuria, representing the White Flag sign. (**B**) A patient with confirmed infective endocarditis demonstrating a definite vegetation on transthoracic echocardiography (yellow arrow). Routine urinalysis showed hematuria and proteinuria, and therefore did not meet the White Flag sign

Between January 2010 and December 2024, we retrospectively screened consecutive adult patients (aged ≥ 19 years) whose echocardiography reports contained the term “vegetation” at a single tertiary center. The corresponding echocardiographic images were independently reviewed by two experienced imaging cardiologists to confirm the presence of vegetation-like findings, defined as mobile, echogenic, mass-like structures attached to endocardial surfaces within the cardiac chambers (Fig. 1). Disagreements between the reviewers were resolved by consensus. Following this review, 574 patients with vegetation-like findings were identified for further clinical evaluation. Transthoracic echocardiography (TTE) served as the primary imaging modality for screening and image review, whereas transesophageal echocardiography (TEE) was performed in selected patients according to the treating physician’s clinical judgment. Each patient underwent comprehensive review of the electronic medical records, including discharge diagnoses (ICD-10 codes I33, I38, and I39), blood culture results, antibiotic treatment, and clinical follow-up. Final IE diagnoses were adjudicated according to the 2023 Duke–ISCVID criteria after integrating the clinical, microbiological, and echocardiographic findings. When available, TEE findings were incorporated into the overall clinical assessment during adjudication. Because of the retrospective study design, final IE adjudication was not performed blinded to urinalysis findings. Patients classified as definite or possible IE were assigned to the IE group, whereas all remaining patients were classified as the non-IE group [11]. At our institution, urinalysis is routinely included in the initial laboratory evaluation of hospitalized patients and is generally performed at the time of admission rather than being selectively ordered based on suspicion of IE. In the outpatient setting, urinalysis was obtained at the discretion of the treating physician as part of the diagnostic evaluation. To ensure that urinalysis reflected the same clinical episode as the echocardiographic findings, the initial urinalysis was defined as a urinalysis performed within 48 h before or after echocardiography. In routine clinical practice, urinalysis was generally performed as part of the initial diagnostic evaluation, typically before or concurrently with blood culture collection. Urinalyses obtained after initiation of antibiotic therapy were not specifically excluded. Patients without an eligible initial urinalysis within this predefined time window were excluded from the final analysis. The characteristics of patients included in the final analysis and those excluded because of unavailable eligible initial urinalysis are summarized in Supplementary Table S1.

**Fig. 2:**
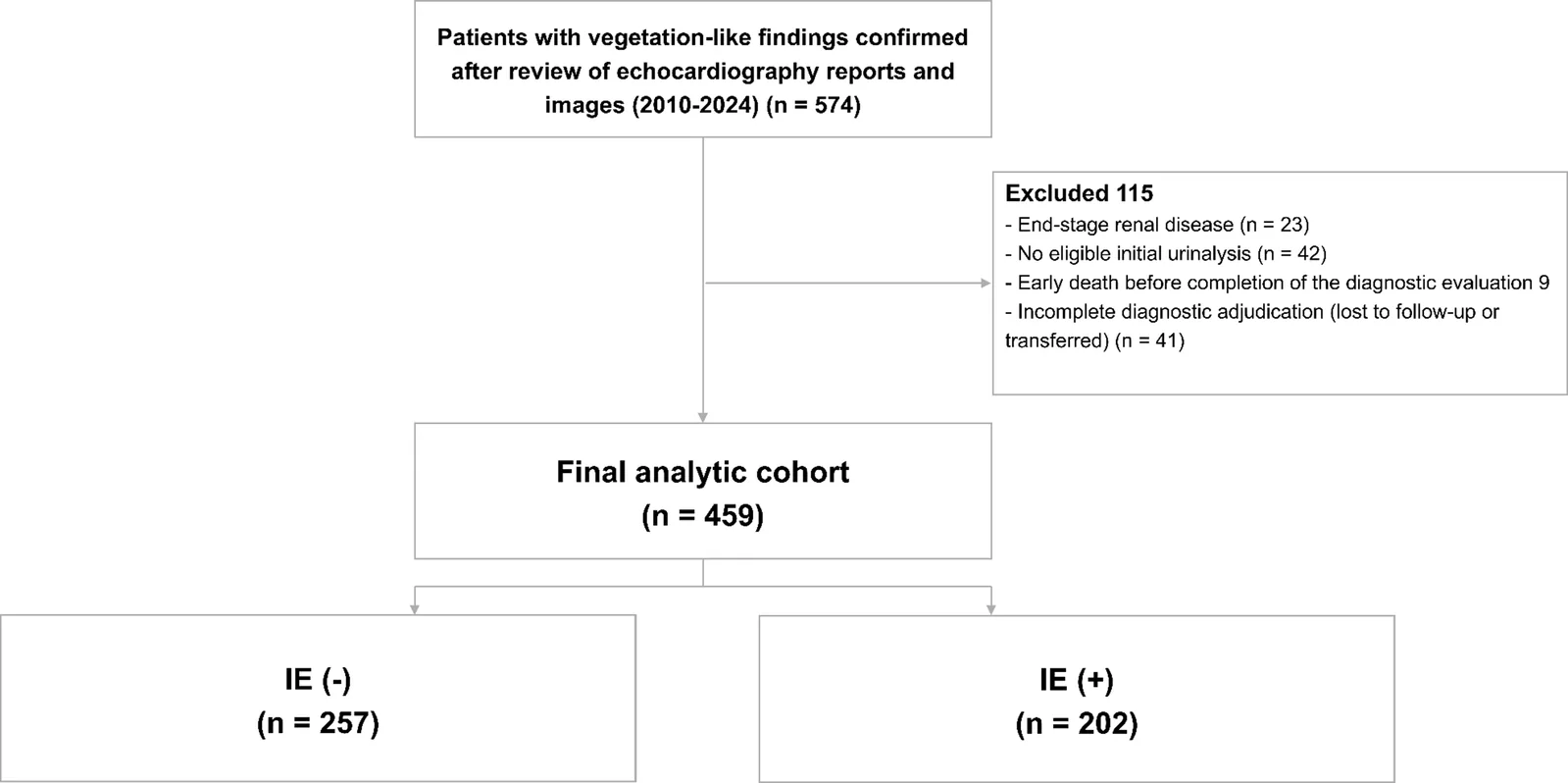
Study flow diagram. Of 574 patients with vegetation-like echocardiographic findings, 115 were excluded according to the predefined criteria, resulting in a final analytic cohort of 459 patients (202 IE [+] and 257 IE [–]). IE, infective endocarditis

Patients were classified into two groups: those with confirmed IE (IE (+); comprising both definite and possible IE by the 2023 Duke–ISCVID criteria) and those with vegetation-like lesions in whom IE was ultimately excluded (IE (–)). Patients with end-stage renal disease were excluded because advanced renal failure may independently affect urinalysis findings [16, 17]. In addition, patients in whom a definitive diagnosis of IE could not be established because of incomplete diagnostic adjudication, including early death or transfer before completion of the diagnostic evaluation, were excluded. A total of 459 patients were included in the final analysis, comprising 202 patients with confirmed IE (139 definite and 63 possible IE) and 257 patients without IE (Fig. 2). The requirement for informed consent was waived by the Institutional Review Board of Gachon University Gil Medical Center (IRB No. GAIRB2026-074) because of the retrospective nature of the study. The study was conducted in accordance with the principles outlined in the Declaration of Helsinki.

### Data collection

Demographic characteristics and clinical information at the time of TTE were extracted from the electronic medical records. Data extraction was performed independently by two investigators. Any uncertain or discrepant information was jointly reviewed and resolved by consensus.

Hypertension was defined as a documented physician diagnosis of hypertension or current use of antihypertensive medication, in accordance with contemporary hypertension guidelines [18]. Diabetes mellitus was defined as a documented physician diagnosis of diabetes mellitus or current use of glucose-lowering medication, according to contemporary diabetes guidelines [19]. Non-dialytic chronic kidney disease (CKD) was defined as an estimated glomerular filtration rate < 60 mL/min/1.73 m² persisting for at least 3 months in patients not receiving renal replacement therapy, according to the Kidney Disease: Improving Global Outcomes (KDIGO) guideline [20]. Other comorbid conditions—including prior stroke or transient ischemic attack, chronic obstructive pulmonary disease or asthma, malignancy, use of immunosuppressive therapy, congenital heart disease (CHD), congestive heart failure (CHF), coronary artery disease (CAD), connective tissue disease (CTD), and liver disease—were identified based on documented medical history and physician diagnosis. Medications that could potentially influence urinalysis findings, including diuretics and renin–angiotensin system inhibitors, were also recorded.

Functional status at initial presentation was assessed using the New York Heart Association (NYHA) functional classification, which categorizes patients according to the severity of symptoms and the degree of limitation of physical activity resulting from cardiac [21]. Laboratory parameters and urinalysis findings obtained at the initial evaluation were recorded. Echocardiographic characteristics associated with susceptibility to IE, including underlying valvular or structural abnormalities and the presence of intracardiac prosthetic material or cardiac implantable electronic devices, were also assessed.

### Urinalysis assessment

The initial urinalysis was performed using standard automated dipstick testing and microscopic examination according to institutional protocols. Hematuria, proteinuria, and pyuria were assessed. For the additive urinalysis score and multivariable analyses, hematuria was defined as dipstick blood positivity (≥ 1+), proteinuria as dipstick protein positivity (≥ 1+), and pyuria as ≥ 6 white blood cells (WBC) per high-power field (HPF). Although microscopic hematuria is conventionally defined as ≥ 3 red blood cells (RBC) per HPF and was also recorded, dipstick blood positivity was selected for the score and regression model to provide a simple and readily applicable tool based on routine urinalysis in everyday clinical practice. Microscopic RBC counts are presented in Table 2 for descriptive comparison only [22–25]. Pyuria was defined as ≥ 6 WBC/HPF on urine microscopy because microscopic WBC counts were more consistently available than dipstick leukocyte esterase results in routine urinalysis reports and therefore provided a more complete measure of pyuria across the study cohort.

### Statistical analysis

Categorical variables are presented as numbers and percentages and were compared using the chi-square test. Continuous variables are expressed as mean ± standard deviation (SD) and were compared using the Student’s *t* test or the Mann–Whitney *U* test, as appropriate. Missing values were not imputed. Analyses were performed using the available data for each variable, and the denominator therefore varied according to data availability where applicable. Multivariable logistic regression analysis was performed to identify urinalysis variables independently associated with IE in patients with vegetation-like echocardiographic findings. Based on these results, a simple additive urinalysis score was constructed by assigning one point to each independently associated urinalysis abnormality. To evaluate the impact of equal weighting, the simple additive score was compared with a logistic regression–based predicted-probability model using the same variables. To assess whether routine urinalysis provided incremental diagnostic information beyond inflammatory markers, three logistic regression models were compared: (1) a urinalysis model(2), an inflammatory marker model including WBC count and C-reactive protein (CRP), and (3) a combined model incorporating both urinalysis variables and inflammatory markers. Diagnostic performance was assessed using the area under the receiver operating characteristic curve (AUC). Internal validation of the urinalysis score was performed using bootstrap resampling with 2,000 repetitions. Model optimism was estimated by comparing discrimination in bootstrap samples with that in the original dataset, and an optimism-corrected AUC was calculated. As a sensitivity analysis, 10-fold cross-validation was also performed. Sensitivity and specificity were calculated for the urinalysis score. All tests were two-sided, and a *P* value < 0.05 was considered statistically significant. Statistical analyses were performed using SPSS (IBM Corp., Armonk, NY, USA), GraphPad Prism version 11 (GraphPad Software, San Diego, CA, USA), and R (R Foundation for Statistical Computing, Vienna, Austria).

## Results

### Baseline characteristics

The baseline characteristics of the participants are presented in Table 1. Mean age was 64 years and 252 patients (54.9%) were men. IE (+) were younger than IE (-) (61 vs. 66 years old, *p* < 0.01). There were no significant differences between groups in the prevalence of hypertension, diabetes mellitus, prior stroke, or CKD. Immunosuppression and congenital heart disease were more common in IE (+), whereas congestive heart failure and coronary artery disease were more common in IE (–). NYHA class III or IV symptoms were more common among IE (+). Although the prevalence of rheumatic valve disease and degenerative valve disease did not differ significantly between groups, mitral valve prolapse was more common in IE (-). TEE was performed in 71 of 202 patients with IE (35.1%) and 65 of 257 patients without IE (25.3%). The final diagnoses of patients in the non-IE group are summarized in Supplementary Table S2. The most common diagnoses were ruptured chordae/flail or thickened leaflets, followed by Lambl’s excrescences, cardiac tumors or masses, degenerative valvular lesions, and thrombi.

**Table 1:** Baseline clinical characteristics of patients with or without infective endocarditis

	Total (*N* = 459)		IE (-) (*N* = 257)	IE (+) (*N* = 202)	*P* value
Age (mean$\:\pm\:$SD)	64$\:\pm\:$16		66$\:\pm\:$14	61$\:\pm\:$17	< 0.01
Gender					0.05
Male	252(54.9%)		130(50.6%)	122(60.4%)	
Female	207(45.1%)		127(49.4%)	80(39.6%)	
Comorbidities					
Hypertension	215(46.8%)		123(47.9%)	92(45.5%)	0.69
Diabetes	110(24.0%)		60(23.3%)	50(24.8%)	0.81
Previous stroke or TIA	54(11.8%)		29(11.3%)	25(12.4%)	0.83
COPD or asthma	22(4.8%)		12(4.7%)	10(5.0%)	1.00
Non-dialytic CKD	36(7.8%)		19(7.4%)	17(8.4%)	0.82
Cancer	78(17.0%)		40(15.6%)	38(18.8%)	0.43
Immunosuppression	24(5.2%)		8(3.1%)	16(7.9%)	0.04
Congenital heart disease	20(4.4%)		6(2.3%)	14(6.9%)	0.03
Congestive heart failure	86(18.7%)		60(23.3%)	26(12.9%)	< 0.01
Coronary artery disease	61(13.3%)		44(17.1%)	17(8.4%)	0.01
Connective tissue disease	8(1.7%)		3(1.2%)	5(2.5%)	0.48
Liver disease	44(9.6%)		19(7.4%)	25(12.4%)	0.10
Urinary tract infection	10 (2.1%)		8 (3.1%)	2 (0.9%)	0.20
Indwelling urinary catheter	92 (20.0%)		32 (12.4%)	60 (29.7%)	< 0.01
Prior antibiotic exposure	41 (8.9%)		17 (6.6%)	24 (11.9%)	0.069
NYHA functional class					< 0.01
Class I	134(29.2%)		124(48.2%)	10(5.0%)	
Class II	184(40.1%)		93(36.2%)	91(45.0%)	
Class III	120(26.1%)		35(13.6%)	85(42.1%)	
Class IV	21(4.6%)		5(1.9%)	16(7.9%)	
Medication					
ARB/ACEi	95(20.7%)		69(36.8%)	26(12.9%)	< 0.01
Antithrombotic agent	123(26.8%)		74(28.8%)	49(24.3%)	0.33
Echocardiographic findings					
Rheumatic valve disease		30(6.5%)	14(5.4%)	16(7.9%)	0.38
Degenerative valve disease	48(10.5%)		27(10.5%)	21(10.4%)	1.00
Mitral prolapse	153(33.3%)		124(48.2%)	29(14.4%)	< 0.01
Bicuspid aortic valve	13(2.8%)		3(1.2%)	10(5.0%)	0.03
Bioprosthetic valve	10(2.2%)		2(0.8%)	8(4.0%)	0.05
Mechanical prosthetic valve	14(3.0%)		6(2.3%)	8(4.0%)	0.46
Nonvalvular intracardiac prosthesis		3(0.7%)	1(0.4%)	2(1.0%)	0.83
Intracardiac devices	10(2.2%)		7(2.7%)	3(1.5%)	0.56
Transesophageal echocardiography	136 (29.6%)		65 (25.3%)	71 (35.1%)	0.03

SD, standard deviation; TIA, transient ischemic attack; COPD, chronic obstructive pulmonary disease; CKD, chronic kidney disease; NYHA, New York Heart Association; ACEi, angiotensin-converting enzyme inhibitor; ARB, angiotensin II receptor blocker; IE: infective endocarditis

Antithrombotic agents included antiplatelet agents and anticoagulants

### Microbiologic findings

Among the 202 patients with IE, 144 (71.3%) had positive blood cultures, whereas 58 (28.7%) were classified as culture-negative IE according to the final clinical adjudication. The distribution of causative microorganisms is summarized in Supplementary Table S3. Prior antibiotic exposure before microbiologic evaluation was documented in 24 patients with IE (11.9%) and 17 patients without IE (6.6%) (*P* = 0.069) (Table 1). In the non-IE group, blood cultures were obtained in 65 of 257 patients (25.3%), including 61 patients with negative cultures and 4 patients with positive cultures. None of these patients fulfilled the 2023 Duke–ISCVID criteria for definite or possible IE after comprehensive clinical adjudication.

### Urinalysis and inflammatory markers according to IE status

Urinalysis demonstrated significantly higher rates of proteinuria (61.4% vs. 23.3%, *p* < 0.01), hematuria (RBC ≥ 3/HPF, 75.7% vs. 36.2%; dipstick blood, 55.4% vs. 20.6%; both *p* < 0.01), and pyuria (34.2% vs. 14.0%, *p* < 0.01) among IE (+). Inflammatory markers, including high-sensitivity C-reactive protein, erythrocyte sedimentation rate, and procalcitonin, were also significantly elevated in IE (+) (Table 2).

**Table 2:** Laboratory and urinalysis findings according to infective endocarditis diagnosis

		Total (*N* = 459)	IE (-) (*N* = 257)	IE (+) (*N* = 202)	*P* value
Urinalysis					
Protein (Dipstick ≥ 1+)		204(44.4%)	60(23.3%)	124(61.4%)	< 0.01
Blood (Dipstick ≥ 1+)		165(36.0%)	53(20.6%)	112(55.4%)	< 0.01
RBC (≥ 3 /HPF)		246(53.6%)	93(36.2%)	153(75.7%)	< 0.01
WBC (≥ 6 /HPF)		105(22.9%)	36(14.0%)	69(34.2%)	< 0.01
WBC esterase (Dipstick ≥ 1+)		191(41.6%)	71(27.6%)	120(59.4%)	< 0.01
Laboratory findings (mean$\:\pm\:$SD^§^)					
Hemoglobin (g/dL)		12.1$\:\pm\:$6.5	12.9$\:\pm\:$8.3	11.0$\:\pm\:$2.4	< 0.01
WBC (/mm^3^)		10,079$\:\pm\:$5,756	8,072$\:\pm\:$4,377	12,632$\:\pm\:$6,687	< 0.01
Platelet (x10^3^/mm^3^)		196$\:\pm\:$95	209$\:\pm\:$82	181$\:\pm\:$107	< 0.01
hsCRP (mg/dL)		6.2$\:\pm\:$8.5	2.5$\:\pm\:$5.1	10.9$\:\pm\:$9.6	< 0.01
ESR (mm/hr)		5.7$\:\pm\:$16.4	4.1$\:\pm\:$13.2	7.7$\:\pm\:$19.6	0.02
Procalcitonin (ng/mL)		2.0$\:\pm\:$8.6	0.3$\:\pm\:$3.2	4.0$\:\pm\:$12.2	< 0.01

RBC, red blood cell; HPF, high-power field; WBC, white blood cell; SD, standard deviation; hsCRP, high-sensitivity C-reactive protein; ESR, erythrocyte sedimentation rate; IE: infective endocarditis

### Multivariable analysis of factors associated with IE

In multivariable logistic regression analysis, younger age (adjusted OR 0.98, 95% CI 0.97–1.00), hematuria (dipstick blood, adjusted OR 2.47, 95% CI 1.52–4.03), proteinuria (adjusted OR 2.56, 95% CI 1.59–4.13), and pyuria (adjusted OR 7.87, 95% CI 3.61–17.14) were independently associated with IE (Table 3).

**Table 3:** Logistic regression analysis of factors associated with infective endocarditis

	Univariate		Multivariate	
	OR (95% CI)	*P*-value	OR (95% CI)	*P*-value
Age at diagnosis	0.98 (0.97–0.99)	0.002	0.98 (0.97–0.99)	0.006
Male	0.95 (0.78–1.17)	0.630	-	-
Immunosuppression	2.32 (0.95–5.64)	0.064	-	-
Congenital heart disease	3.12 (1.18–8.26)	0.022	-	-
Urinalysis Parameters				
Blood (≥ 1+)	4.79 (3.18–7.22)	< 0.001	2.47 (1.52–4.03)	< 0.001
Protein (≥ 1+)	5.22 (3.50–7.87)	< 0.001	2.56 (1.59–4.13)	< 0.001
WBC (≥ 6/HPF)	14.30 (6.92–29.54)	< 0.001	7.87 (3.61–17.14)	< 0.001

OR, odds ratio; CI, confidence interval; WBC, white blood cell; HPF, high-power field

### Urinalysis-based model

**Fig. 3:**
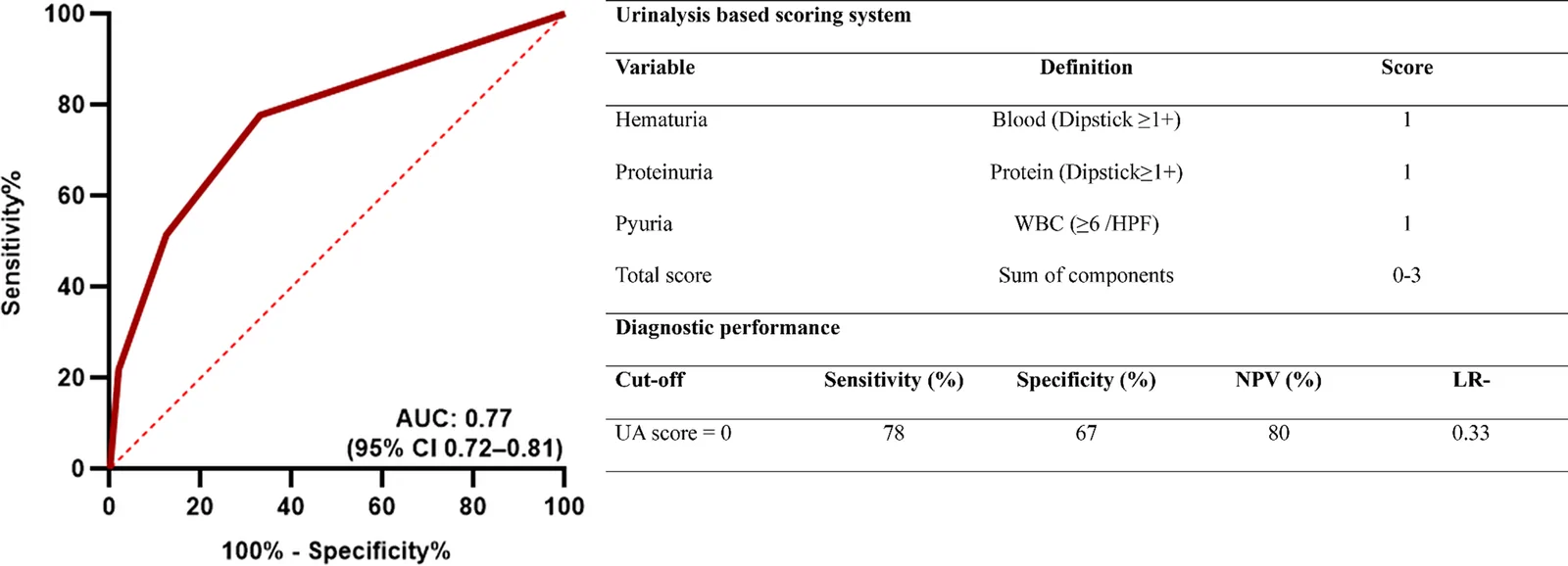
Diagnostic performance of the urinalysis-based scoring system for infective endocarditis. ROC curve demonstrating the diagnostic performance of the additive urinalysis score based on dipstick hematuria (blood ≥ 1+), dipstick proteinuria (protein ≥ 1+), and pyuria (≥ 6 WBC/HPF). One point was assigned for each abnormal finding (total score range, 0–3). A score of 0 demonstrated a sensitivity of 78%, specificity of 67%, negative predictive value of 80%, and negative likelihood ratio of 0.33 for infective endocarditis. The score is intended as a supportive adjunct to clinical assessment rather than a standalone rule-out test. ROC, receiver operating characteristic; AUC, area under the curve; CI, confidence interval; UA, urinalysis; WBC, white blood cell; HPF, high-power field; NPV, negative predictive value; LR−, negative likelihood ratio

A simple urinalysis-based additive score was constructed by assigning one point each for the presence of hematuria, proteinuria, and pyuria (total score range, 0–3). The model demonstrated moderate discriminatory ability, with an AUC of 0.77 (95% CI, 0.71–0.80). In this selected population, a score of 0 yielded a sensitivity of 78%, a specificity of 67%, a negative predictive value of 80%, and a negative likelihood ratio of 0.33. However, 22% of patients with IE had a score of 0, indicating that the score lowers, but does not exclude, the likelihood of IE (Fig. 3).

To further evaluate the performance of the score in patients with lower clinical suspicion for IE, an additional subgroup analysis was performed in afebrile patients. Among 277 afebrile patients, 47 (17.0%) were ultimately diagnosed with IE. The urinalysis-based score maintained good discriminatory performance, with an AUC of 0.78 (95% CI, 0.70–0.86). Using a score threshold of ≥ 1, the sensitivity was 77.7% and the specificity was 66.9% (Supplementary Figure S1).

To assess whether weighting the individual urinalysis variables improved performance, the simple additive score was compared with a logistic regression–based predicted-probability model using the same variables. The two approaches demonstrated very similar discrimination (AUC, 0.769 vs. 0.776), supporting the use of the simpler additive score for routine clinical application (Supplementary Table S4).

Internal validation using 2,000 bootstrap resamples demonstrated minimal optimism (mean optimism, 0.0002). The optimism-corrected AUC was 0.77, which was nearly identical to the apparent AUC of 0.77. Similarly, 10-fold cross-validation yielded a mean AUC of 0.77 (SD, 0.073), indicating stable model performance with little evidence of overfitting (Supplementary Table S5).

### Exploratory comparison with inflammatory marker models

To explore the incremental diagnostic value of routine urinalysis beyond inflammatory markers, we compared three logistic regression models: a urinalysis model (dipstick blood positivity, proteinuria, and pyuria), an inflammatory marker model (WBC count and CRP), and a combined model including both urinalysis variables and inflammatory markers. The urinalysis model demonstrated moderate discrimination (AUC, 0.78; 95% CI, 0.73–0.82), whereas the inflammatory marker model showed better discrimination (AUC, 0.82; 95% CI, 0.78–0.86). The combined model further improved discrimination (AUC, 0.85; 95% CI, 0.81–0.89), suggesting that urinalysis may provide complementary diagnostic information beyond nonspecific inflammatory markers (Supplementary Figure S2).

## Discussion

### Principal findings

In this study, urinalysis provided a simple and sensitive adjunctive marker to support clinical assessment in patients with vegetation-like echocardiographic findings. Hematuria, proteinuria, and pyuria were significantly more common in IE (+) than in IE (–). A urinalysis-based approach demonstrated acceptable discriminatory ability, and the absence of urinary abnormalities was associated with a lower likelihood of IE in this selected population.

### Clinical challenge in differentiating vegetation-like echocardiographic findings

Despite its established role as a cornerstone imaging modality for IE, echocardiography has inherent limitations in differentiating true vegetation from non-infectious intracardiac findings, particularly evident in the early or subacute stages of the disease and in patients with intracardiac materials such as prosthetic valves or cardiac devices [4, 26]. Non-infectious findings may mimic vegetation, leading to diagnostic uncertainty and delayed clinical decision-making, even when IE is ultimately excluded [3]. In our cohort, the most common non-IE diagnoses were abnormalities of the mitral valve apparatus, including ruptured chordae, flail or thickened leaflets, followed by Lambl’s excrescences, cardiac masses, degenerative valvular lesions, and thrombi (Supplementary Table S2). These findings illustrate the broad spectrum of non-infectious conditions that may mimic IE and contribute to diagnostic uncertainty. Additional readily available clinical information may assist early diagnostic assessment. Routine urinalysis is an inexpensive, noninvasive test that can be performed rapidly in both inpatient and outpatient settings. Although it cannot establish or exclude the diagnosis of IE on its own, our findings suggest that urinalysis may serve as a practical adjunct to clinical, microbiological, and imaging assessment by helping to stratify the likelihood of IE in patients with vegetation-like echocardiographic findings.

### Rationale for urinalysis in early clinical assessment of IE

One potential explanation for the diagnostic value of urinalysis observed in our study lies in the characteristic renal involvement of IE. Renal manifestations of IE are multifactorial and are commonly mediated by immune complex deposition, complement activation, and systemic inflammatory responses, resulting in glomerular injury and urinary abnormalities such as hematuria, proteinuria, and pyuria [14, 15, 27]. In addition, septic embolization and renal microvascular involvement may further contribute to these urinary findings. These findings may occur even without overt renal dysfunction and are detectable on routine urinalysis [28, 29]. 

Although urinalysis is recognized as part of the minor diagnostic criteria for IE in the 2023 ESC guidelines [11], its potential contribution to early clinical assessment has received relatively little attention. Individual urinary abnormalities are nonspecific and have limited diagnostic value when interpreted in isolation [30]. However, rather than reflecting a single pathological process, the combined presence of hematuria, proteinuria, and pyuria may represent the cumulative effects of immune-mediated glomerular injury, systemic inflammation, and renal microvascular involvement in IE, thereby providing a more informative clinical signal than any individual urinalysis parameter alone. This interpretation is supported by previous studies demonstrating that hematuria reflects clinically relevant renal involvement and may provide incremental diagnostic value in the evaluation of IE [31, 32]. Conversely, the absence of these urinary abnormalities may indicate a lower likelihood of such underlying pathological processes, which may explain the high sensitivity of our simple urinalysis-based score.

Because urinalysis is inexpensive, noninvasive, rapidly obtainable, and routinely performed at the initial evaluation of patients with suspected infection, it represents a practical adjunct to early clinical assessment. Rather than serving as a standalone diagnostic test, urinalysis may complement clinical evaluation, microbiological investigations, and imaging findings to support early risk stratification in patients with vegetation-like echocardiographic findings, particularly while definitive diagnostic information is still being obtained [33]. Although urinary abnormalities are nonspecific and may also be associated with urinary tract infection, chronic kidney disease, or other inflammatory conditions, our approach does not rely on any single abnormal finding but rather on their combined pattern and, importantly, their absence.

### Comparison with previous studies

Previous studies have recognized urinary abnormalities as manifestations of the renal and immunological involvement of IE. Microscopic hematuria and proteinuria have long been described in association with immune complex-mediated glomerulonephritis and renal embolic phenomena in IE, supporting the biological plausibility of routine urinalysis as part of the diagnostic evaluation [14, 15, 28]. More recently, systematic assessment of immunological phenomena, including hematuria, has been shown to improve the classification of patients with suspected IE, increasing the proportion of cases classified as definite IE and supporting the value of routinely evaluating these findings during the diagnostic work-up [31]. imilarly, another recent study proposed microhematuria as a potential additional minor diagnostic criterion after multivariable modeling, suggesting that it may provide incremental diagnostic information when interpreted alongside the current Duke criteria [32]. 

Our findings extend these observations in several important respects. Rather than evaluating urinary abnormalities solely as manifestations of established IE or focusing on microhematuria alone, we investigated the diagnostic utility of routine urinalysis in patients presenting with vegetation-like echocardiographic findings, a population in whom diagnostic uncertainty is common. Furthermore, we evaluated the combined pattern of hematuria, proteinuria, and pyuria rather than individual urinary abnormalities, emphasizing that the absence of these findings may help identify patients with a relatively lower likelihood of IE. Taken together, these findings suggest that routine urinalysis may be most valuable not as an isolated diagnostic criterion, but as a simple and readily available adjunct to clinical, microbiological, and imaging assessment during the early evaluation of suspected IE.

### Practical strengths of the “Triple White Flag” approach

Our study has several practical strengths. Rather than developing a complex prediction model, we evaluated routinely performed urinalysis in a clinically relevant population with vegetation-like echocardiographic findings and developed the simple “Triple White Flag” score based on the absence of hematuria, proteinuria, and pyuria. The score was intentionally designed to prioritize simplicity and bedside applicability over maximal statistical performance. Although pyuria showed a stronger association with IE than the other urinalysis variables, weighted scoring yielded only minimal improvement over the simple additive model, supporting the use of an equal-weight score in routine practice. Likewise, while inflammatory markers demonstrated better discrimination than urinalysis alone, the addition of routine urinalysis provided modest incremental diagnostic value, suggesting that urinary abnormalities capture complementary information beyond systemic inflammation. Together, these findings support the potential role of the Triple White Flag score as a practical adjunct during the initial evaluation of patients with suspected IE, although prospective external validation remains necessary.

### Limitations

Several limitations should be considered when interpreting our findings. First, this was a retrospective single-center study, and residual confounding cannot be excluded. Urinary abnormalities may have been influenced by factors such as urinary tract infection, chronic kidney disease, urinary catheterization, anticoagulant use, and other clinical conditions that were not uniformly captured because of the retrospective design. In addition, urinary symptoms, menstruation or other gynecologic sources of bleeding, and urine culture results were not systematically available because of the retrospective study design. Because final IE adjudication was performed retrospectively and was not blinded to urinalysis findings, diagnostic review bias cannot be completely excluded.

Second, the study population was restricted to patients with vegetation-like echocardiographic findings at a tertiary referral center, representing a selected population undergoing evaluation for possible IE. Therefore, the generalizability of our findings to other clinical settings, such as emergency department or outpatient populations, remains uncertain.

Third, although the White Flag sign demonstrated stable internal performance, it was derived and internally validated within the same cohort and has not undergone external validation. Prospective multicenter studies are needed to confirm its diagnostic performance, clinical utility, and potential impact on diagnostic pathways.

Finally, detailed echocardiographic characteristics, including vegetation size, mobility, and image quality, were not systematically recorded, and formal assessment of interobserver agreement was not performed. TEE was also not performed according to a standardized study protocol, precluding assessment of its independent contribution to the final diagnosis. In addition, microbiological subgroup analyses were exploratory because the study was not designed or sufficiently powered to evaluate pathogen-specific differences in urinalysis findings.

## Conclusions

In conclusion, the absence of hematuria, proteinuria, and pyuria (“white flag sign”) was associated with a lower likelihood of IE in patients with vegetation-like echocardiographic findings. Although it should not be considered a rule-out test, this simple approach may serve as a practical adjunct to clinical, microbiological, and echocardiographic assessment during the early evaluation of suspected IE. Prospective external validation is required before broader clinical implementation.

## Supplementary Information

Below is the link to the electronic supplementary material.

Supplementary Material 1

Supplementary Material 2

## Data Availability

The datasets generated and/or analysed during the current study are not publicly available due to institutional and patient privacy restrictions but are available from the corresponding author on reasonable request.

## Abbreviations

ACEi: Angiotensin-converting enzyme inhibitor
ARB: Angiotensin II receptor blocker
AUC: Area under the receiver operating characteristic curve
CAD: Coronary artery disease
CHD: Congenital heart disease
CHF: Congestive heart failure
CI: Confidence interval
CKD: Chronic kidney disease
COPD: Chronic obstructive pulmonary disease
CTD: Connective tissue disease
EMR: Electronic medical records
ESR: Erythrocyte sedimentation rate
HPF: High-power field
hsCRP: High-sensitivity C-reactive protein
ICD-10: International Classification of Diseases, 10th Revision
IE: Infective endocarditis
IRB: Institutional Review Board
ISCVID: International Society for Cardiovascular Infectious Diseases
NYHA: New York Heart Association
OR: Odds ratio
RBC: Red blood cells
SD: Standard deviation
TIA: Transient ischemic attack
TTE: Transthoracic echocardiography
WBC: White blood cells
