# Supplementary material for: Routine urinalysis for early clinical assessment in patients with vegetation-like echocardiographic findings
Source: BMC Infect Dis. 2026 Jul 23;26:1679. doi: 10.1186/s12879-026-14072-1 (PMC13589381; doi:10.1186/s12879-026-14072-1)
Supplement: Supplementary file 1 [file 12879_2026_14072_MOESM1_ESM.docx]

**Supplementary Table S1. Comparison of patients included in the final analysis and those excluded because of unavailable eligible initial urinalysis**

|  | **Included in the final analysis (n = 459)** | **Excluded because of unavailable eligible initial urinalysis (n = 42)** | **P value** |
| --- | --- | --- | --- |
| Age, years | 64±16 | 63.3±11 | 0.707 |
| Male sex | 252 (54.9%) | 22 (52.4%) | 0.749 |
| Hospitalized at the index evaluation | 411 (89.5%) | 13 (30.9%) | <0.001 |
| Blood culture performed | 267 (58.2%) | 5 (11.9%) | <0.001 |
| TEE performed | 130 (28.3%) | 1 (2%) | <0.001 |

UA, urinalysis; TEE, transesophageal echocardiography

**Supplementary Table S2. Final echocardiographic diagnoses in patients without infective endocarditis**

| **Echocardiographic final diagnosis** | **n** | **% of IE (–)** |
| --- | --- | --- |
| Chordae rupture / flail leaflet | 151 | 59% |
| Lambl’s excrescence | 21 | 8% |
| Cardiac tumor / mass | 15 | 6% |
| Degenerative / thickened valve | 14 | 5% |
| Thrombus | 9 | 4% |
| Prosthetic material / device | 4 | 2% |
| Nonbacterial thrombotic endocarditis | 3 | 1% |
| Mitral valve prolapse | 2 | 1% |
| Other / not otherwise specified | 38 | 15% |

IE, infective endocarditis

**Supplementary Table S3. Urinalysis findings according to the microbiological etiology of infective endocarditis**

| **Organism** | **n (%)** | **Hematuria** | **Proteinuria** | **Pyuria** |
| --- | --- | --- | --- | --- |
| Streptococci | 47 (23%) | 72% | 51% | 13% |
| Staphylococcus aureus | 35 (17%) | 89% | 83% | 49% |
| Enterococci | 20 (10%) | 70% | 50% | 40% |
| Other staphylococci | 15 (7%) | 67% | 53% | 47% |
| Other organisms | 27 (13%) | 74% | 67% | 44% |
| Culture-negative | 58 (29%) | 76% | 60% | 33% |

**Supplementary Table S4. Comparison of the simple additive urinalysis score and the logistic regression–based predicted-probability model**

| **Model** | **Description** | **AUC (95% CI)** |
| --- | --- | --- |
| Simple additive score | 1 point each for dipstick blood positivity, proteinuria, and pyuria | 0.77 (0.72–0.81) |
| Logistic regression–based predicted-probability model | Weighted coefficients for the same three urinalysis variables | 0.78 (0.73–0.82) |

AUC, area under the receiver operating characteristic curve; CI, confidence interval.

**Supplementary Table S5. Internal validation of the urinalysis-based additive score**

| **Metric** | **Value** |
| --- | --- |
| Apparent AUC | 0.77 |
| Mean optimism | 0.0002 |
| Optimism-corrected AUC | 0.77 |
| Bootstrap 95% CI | 0.73–0.81 |
| 10-fold cross-validation mean AUC (SD) | 0.77 (0.073) |
| Bootstrap repetitions | 2,000 |

AUC, area under the receiver operating characteristic curve; CI, confidence interval; SD, standard deviation.
