# Supplementary material for: Routine urinalysis for early clinical assessment in patients with vegetation-like echocardiographic findings
Source: BMC Infect Dis. 2026 Jul 23;26:1679. doi: 10.1186/s12879-026-14072-1 (PMC13589381; doi:10.1186/s12879-026-14072-1)
Supplement: Supplementary file 2 [file 12879_2026_14072_MOESM2_ESM.docx]

**Supplementary Figure 1. Receiver operating characteristic (ROC) curve of the urinalysis-based score in afebrile patients.**

ROC curve demonstrating the diagnostic performance of the urinalysis-based additive score in afebrile patients. The AUC was 0.78 (95% CI, 0.70–0.86).

ROC, receiver operating characteristic; AUC, area under the receiver operating characteristic curve; CI, confidence interval.

**Supplementary Figure 2. Comparison of the diagnostic performance of the urinalysis model, inflammatory marker model, and combined model.**

Receiver operating characteristic (ROC) curves comparing the diagnostic performance of the urinalysis model (dipstick blood positivity, proteinuria, and pyuria), the inflammatory marker model (white blood cell count and high-sensitivity C-reactive protein), and the combined model incorporating both urinalysis and inflammatory marker variables. The combined model demonstrated the highest discrimination (AUC, 0.85; 95% CI, 0.81–0.89). These analyses were exploratory and were performed to evaluate the incremental diagnostic contribution of routine urinalysis beyond inflammatory markers.

ROC, receiver operating characteristic; AUC, area under the receiver operating characteristic curve; CI, confidence interval; WBC, white blood cell; CRP, C-reactive protein.
